# Supplementary material for: A rose flavor compound activating the NRF2 pathway in dendritic cells ameliorates contact hypersensitivity in mice
Source: Front Nutr. 2023 Feb 9;10:1081263. doi: 10.3389/fnut.2023.1081263 (PMC9946980; doi:10.3389/fnut.2023.1081263)
Supplement: Supplementary file 1 [file Data_Sheet_1.docx]

Supplementary Material

**Supplementary Table 1.** The information of primers used in quantitative PCR.

| Primer | Sequence |
| --- | --- |
| mIl6_F | AATCGTGGAAATGAGAAAAGAGTTG |
| mIl6_R | AGTGCATCATCGTTGTTCATACAA |
| mTnf_F | AGGGATGAGAAGTTCCCAAATG |
| mTnf_R | TGTGAGGGTCTGGGCCATA |
| mGapdh_F | ACGTGCCGCCTGGAGAA |
| mGapdh_R | GATGCCTGCTTCACCACCTT |
| mIl12b_F | GAAGCACGGCAGCAGAATAAA |
| mIl12b_R | GGTTTGATGATGTCCCTGATGA |
| mIl23a_F | ATCCAGTGTGAAGATGGTTGTGA |
| mIl23a_R | CGGATCCTTTGCAAGCAGAA |
| mHmox1_F | CACAGGGTGACAGAAGAGGCTAA |
| mHmox1_R | CAGCTCCTCAAACAGCTCAATG |
| mHqo1_F | TGGCCGAACACAAGAAGCT |
| mHqo1_R | CACTGCAATGGGAACTGAAATATC |

**Supplementary Table 2.** A list of compounds in an aroma chemical library.

| Compound | MW (g/mol) | Density (g/mL) |
| --- | --- | --- |
| Furfural | 96.09 | 1.16 |
| 2-Piperonylpropanal | 192.21 | 1.16 |
| Salicylaldehyde | 122.12 | 1.15 |
| Benzyl alcohol | 108.14 | 1.05 |
| Furfuryl alcohol | 98.10 | 1.13 |
| 3-Methylnonane-2,4-dione | 170.25 | 0.90 |
| Triacetin | 218.20 | 1.16 |
| Isobutyl acetate | 116.16 | 0.87 |
| Butyl butyryllactate | 216.28 | 0.97 |
| Diethyl succinate | 174.20 | 1.05 |
| δ-Dodecanolactone | 198.31 | 0.94 |
| Ethyl acetoacetate | 130.14 | 1.02 |
| Ethyl dodecanoate | 228.38 | 0.86 |
| Ethyl nonanoate | 186.30 | 0.87 |
| Ambrettolide | 252.39 | 0.96 |
| Methyl phenylacetate | 150.18 | 1.07 |
| Triethyl citrate | 276.29 | 1.14 |
| γ-Pentalactone | 100.12 | 1.06 |
| Methyl epi-dihydrojasmonate | 226.32 | 1.00 |
| Hexyl phenylacetate | 220.31 | 0.97 |
| Ethylene brassylate | 270.37 | 1.04 |
| *cis*-3-Hexenyl lactate | 172.22 | 0.98 |
| *cis*-3-Hexenyl pyruvate | 170.21 | 0.99 |
| Menthyl 3-hydroxybutyrate | 242.36 | 0.98 |
| Isoamyl salicylate | 208.26 | 1.05 |
| Benzyl salicylate | 228.24 | 1.18 |
| Allyl cinnamate | 188.22 | 1.05 |
| Allyl phenoxyacetate | 192.21 | 1.10 |
| Furaneol acetate | 170.16 | 1.17 |
| Mintlactone | 166.22 | 1.06 |
| Isoamyl alcohol | 88.15 | 0.81 |
| Anisole | 108.14 | 1.00 |
| Benzaldehyde propyleneglycol acetal | 164.20 | 1.07 |
| β-Caryophyllene | 204.36 | 0.90 |
| *p*-Cymene | 134.22 | 0.86 |
| Decanol | 158.29 | 0.83 |
| Diacetyl | 86.09 | 0.98 |
| 1,8-Cineole | 154.25 | 0.92 |
| Acetic acid | 60.05 | 1.05 |
| Hexanoic acid | 116.16 | 0.93 |
| Compound | MW (g/mol) | Density (g/mL) |
| Hydroxycitronellal diethyl acetal | 230.39 | 0.90 |
| Levulinic acid | 116.12 | 1.13 |
| Nonanoic acid | 158.24 | 0.91 |
| Oleic acid | 282.47 | 0.89 |
| α-Pinene | 136.24 | 0.86 |
| Terpinolene | 136.24 | 0.86 |
| 2-Pentylfuran | 138.21 | 0.89 |
| Bisabolene | 204.35 | 0.86 |
| Hexanal propyleneglycol acetal | 158.24 | 0.90 |
| 1,4-Cineole | 154.25 | 0.90 |
| Menthoxypropanediol | 230.34 | 1.00 |
| 1,3,5-Undecatriene | 150.27 | 0.80 |
| *p*-Menthan-3,8-diol | 172.26 | 0.98 |
| Isovaleraldehyde diethyl acetal | 160.25 | 0.84 |
| Carvacrol | 150.22 | 0.98 |
| 4-Ethylguaiacol | 152.19 | 1.06 |
| Nerolidol | 222.37 | 0.87 |
| Hotrienol | 152.23 | 0.89 |
| α-Hexylcinnamaldehyde | 216.32 | 0.96 |
| *trans*-2-Heptenal | 112.17 | 0.86 |
| 5-Methyl-2-phenyl-2-hexenal | 188.27 | 0.97 |
| 2-Phenylcrotonaldehyde | 146.19 | 1.05 |
| 2-isopropyl-5-methyl-2-hexenal | 154.25 | 0.85 |
| Linalool oxide | 170.25 | 0.94 |
| α-Irone | 206.32 | 0.93 |
| *cis*-Jasmone | 164.25 | 0.94 |
| 4-Oxoisophorone | 152.19 | 1.03 |
| Mesifurane | 142.15 | 1.10 |
| Benzaldehyde | 106.12 | 1.05 |
| Cuminaldehyde | 148.21 | 0.98 |
| Hydroxycitronellal | 172.26 | 0.93 |
| Dodecanal | 184.32 | 0.83 |
| 5-Methyl-2-furfural | 110.11 | 1.11 |
| Capryl aldehyde | 128.22 | 0.82 |
| Anisaldehyde | 136.15 | 1.12 |
| Styrallyl alcohol | 122.17 | 1.01 |
| Ethyl 3-methyl-3-phenylglycidate | 206.24 | 1.09 |
| Amyl hexanoate | 186.30 | 0.87 |
| Anisyl acetate | 180.20 | 1.11 |
| Benzyl benzoate | 212.25 | 1.12 |
| Compound | MW (g/mol) | Density (g/mL) |
| Isobornyl acetate | 196.29 | 0.99 |
| Butyl 10-undecenoate | 240.38 | 0.87 |
| Citronellyl butyrate | 226.36 | 0.87 |
| γ-Decanolactone | 170.25 | 0.95 |
| Diethyl malonate | 160.17 | 1.06 |
| γ-Dodecanolactone | 198.31 | 0.94 |
| Ethyl formate | 74.08 | 0.92 |
| Ethyl lactate | 118.13 | 1.03 |
| Ethyl levulinate | 144.17 | 1.01 |
| Ethyl 2-methylbutyrate | 130.19 | 0.87 |
| Ethyl pyruvate | 116.12 | 1.05 |
| Furfuryl acetate | 140.14 | 1.12 |
| Methyl isovalerate | 116.16 | 0.88 |
| Methyl isobutyrate | 102.13 | 0.89 |
| Methyl heptine carbonate | 154.21 | 0.92 |
| Phenethyl isobutyrate | 192.25 | 0.99 |
| Phenoxyethyl isobutyrate | 208.26 | 1.05 |
| α-Terpinyl acetate | 196.29 | 0.95 |
| Methyl epi-jasmonate | 224.30 | 1.03 |
| Methyl linoleate | 294.48 | 0.89 |
| Ethyl 3-hydroxyhexanoate | 160.21 | 0.98 |
| δ-Tetradecalactone | 226.36 | 0.94 |
| Octahydrocoumarin | 154.21 | 1.09 |
| Linalyl acetate | 196.29 | 0.90 |
| Acetaldehyde diethyl acetal | 118.18 | 0.83 |
| Amyl alcohol | 88.15 | 0.82 |
| Isoeugenyl methyl ether | 178.23 | 1.05 |
| 2,3-Hexanedione | 114.14 | 0.93 |
| Myrcene | 136.24 | 0.80 |
| Nonanol | 144.26 | 0.83 |
| α-Phellandrene | 136.24 | 0.84 |
| β-Pinene | 136.24 | 0.87 |
| Propanol | 60.10 | 0.80 |
| Dihydroanethole | 150.22 | 0.94 |
| Pyruvic acid | 88.06 | 1.27 |
| Methyl 2-phenylethyl ether | 136.19 | 0.95 |
| 2-Methyl-3-oxo-tetrahydrofuran | 100.12 | 1.03 |
| Isomenthone | 154.25 | 0.88 |
| Geranylacetone | 194.32 | 0.87 |
| Diphenyl oxide | 170.21 | 1.08 |
| Compound | MW (g/mol) | Density (g/mL) |
| *cis*-5-Decenoic acid | 170.25 | N/A |
| 1,2-Dimethoxybenzene | 138.16 | 1.08 |
| 3-Carene | 136.23 | 0.86 |
| 2,3-Dihydrofarnesol | 224.39 | 0.87 |
| Decanal | 156.27 | 0.83 |
| Propanal | 58.08 | 0.81 |
| 3,4-Dihydrocoumarin | 148.16 | 1.17 |
| Ethyl oleate | 310.51 | 0.87 |
| γ-Heptanolactone | 128.17 | 1.00 |
| Butyl vanillyl ether | 210.27 | 1.06 |
| Linalool | 154.25 | 0.86 |
| Perillyl alcohol | 152.23 | 0.96 |
| Citral | 152.23 | 0.89 |
| Theaspirane | 194.31 | 0.93 |
| *trans*-2-Hexenal diethyl acetal | 172.27 | 0.85 |
| β-Damascone | 192.30 | 0.93 |
| 8,9-Dehydronootkatone | 216.32 | 1.01 |
| Perillaldehyde | 150.22 | 0.95 |
| Methyl salicylate | 152.15 | 1.19 |
| Ethyl salicylate | 166.18 | 1.13 |
| Isobutyl salicylate | 194.23 | 1.07 |
| Amyl salicylate | 208.25 | 1.06 |
| Prenyl salicylate | 206.24 | 1.15 |
| Hexyl salicylate | 222.28 | 1.04 |
| *cis*-3-Hexenyl salicylate | 220.26 | 1.06 |
| Cyclohexyl salicylate | 220.26 | 1.11 |
| Isopropoxyethyl salicylate | 224.26 | 1.10 |

**Supplementary Figures and Figure Legends**


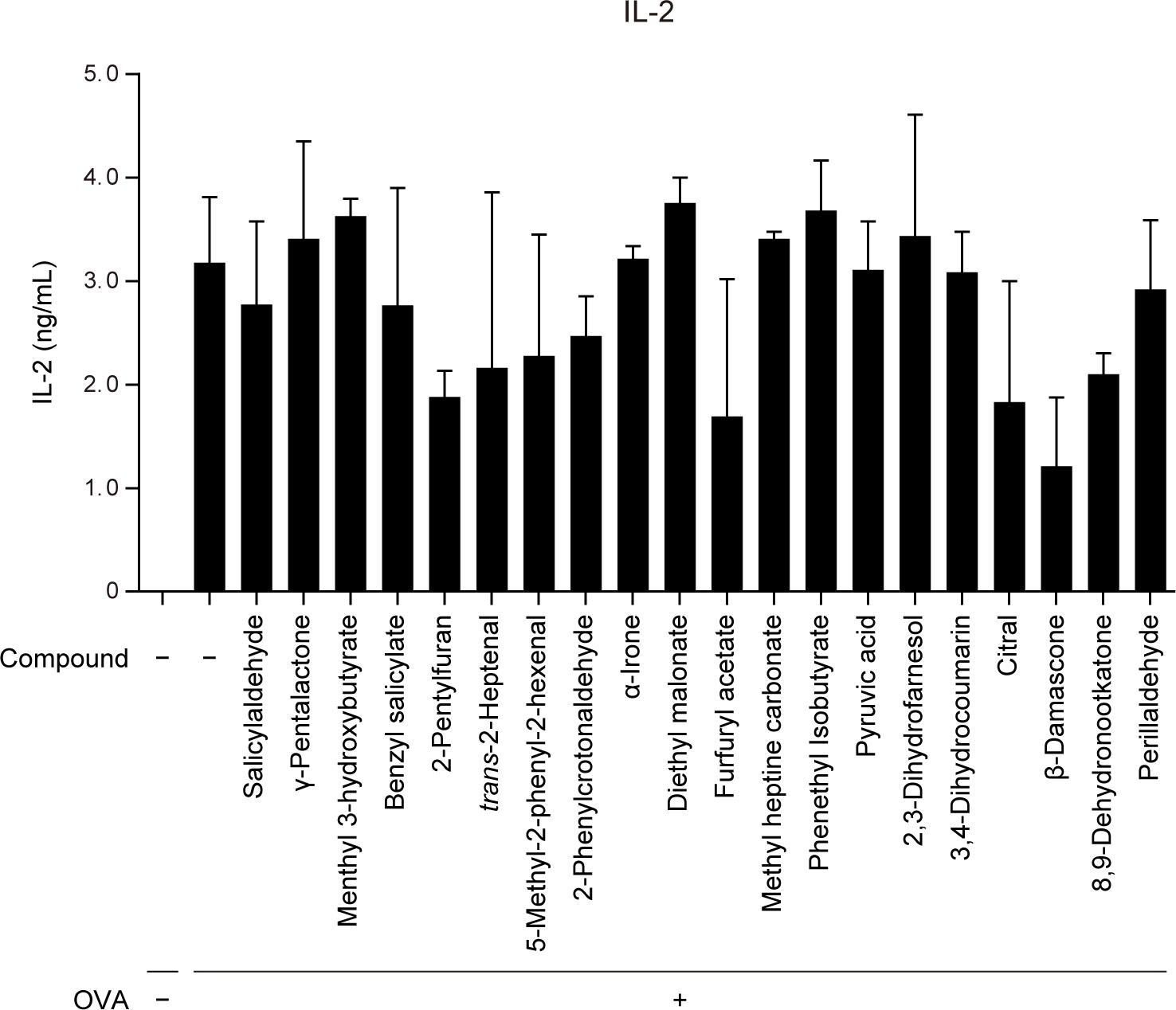


**Supplementary Figure 1.** IL-2 production levels in the second screening.

The numbers of compounds are those in a library list (**Supplementary Table SII**). Each compound was added to the culture media of OVA-pulsed OT-II spleen cells at 0.001% (vol/vol) of the final concentration.


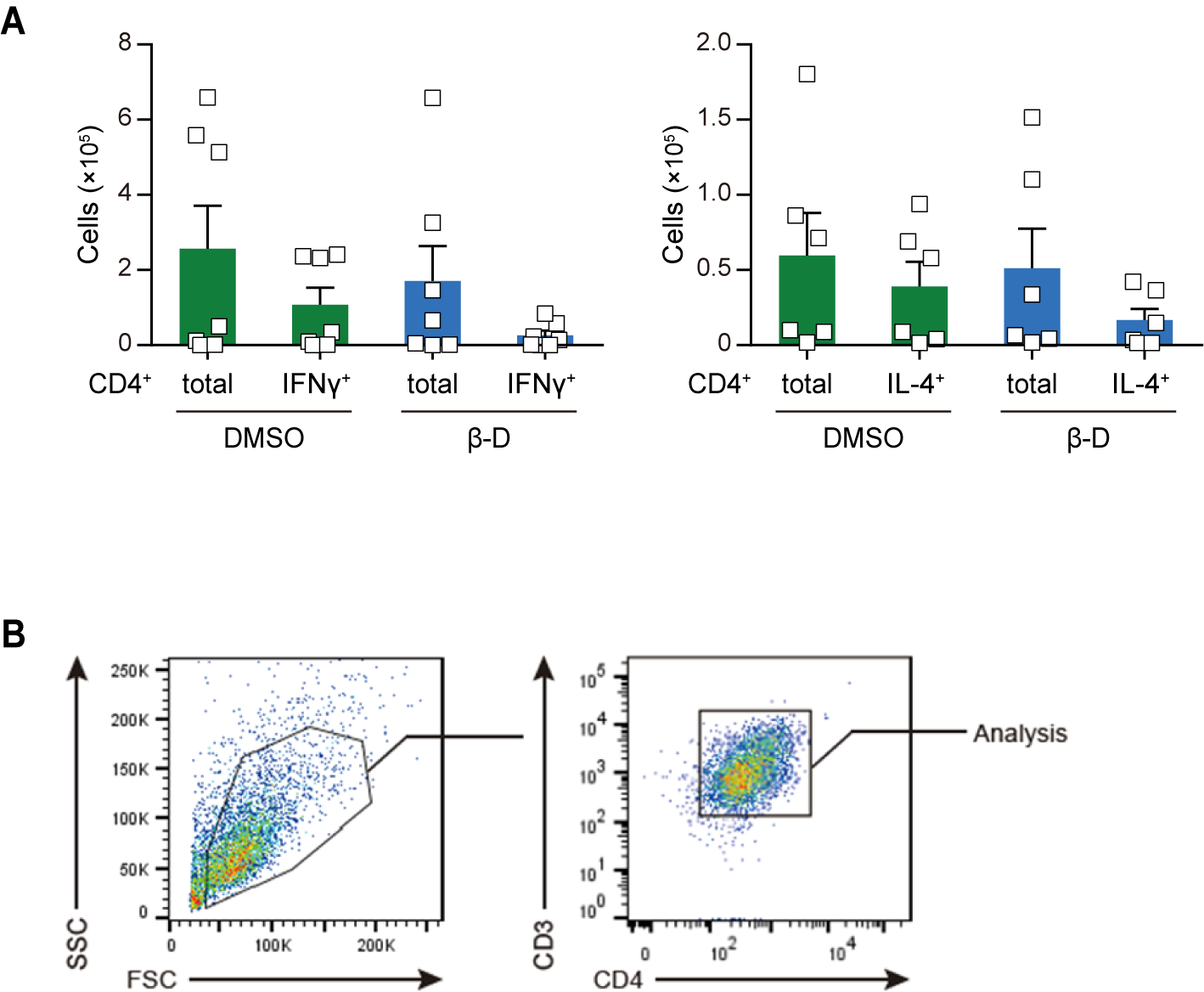


**Supplementary Figure 2.** The numbers of CD4^+^ T cells in Th1- or Th2-polarizing conditions (**A**), and gating strategies of CD4^+^ T cells purified from spleen (**B**).


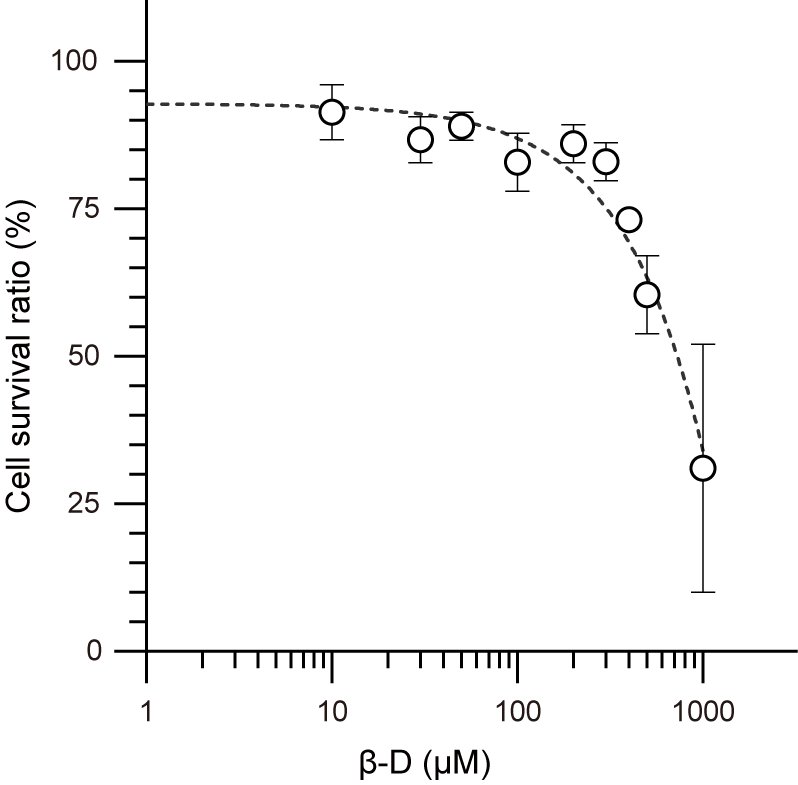


**Supplementary Figure 3.** Cell viabilities of DCs in the presence of β-damascone.

BMDCs were preincubated with the indicated concentrations of β-damascone for 24 h. Cell viability was judged with DAPI staining.

**
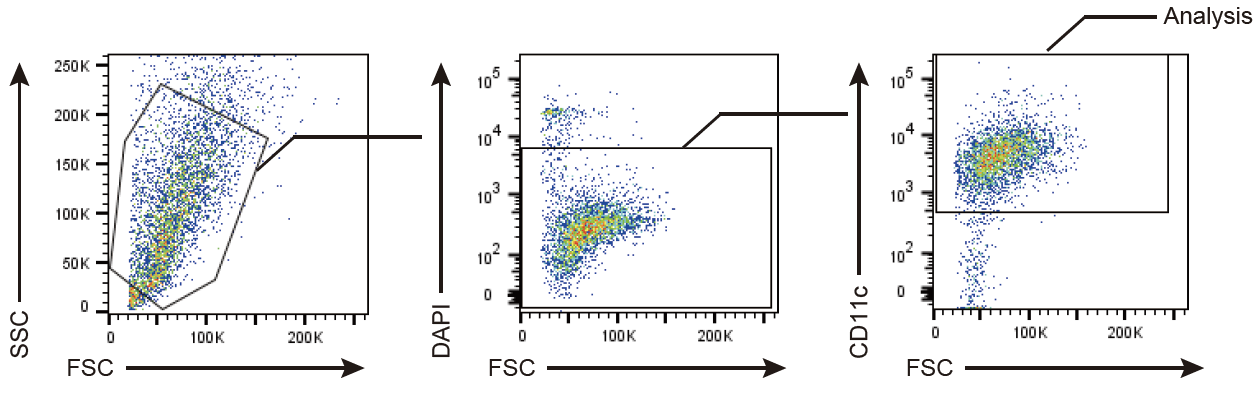
**

**Supplementary Figure 4.** Gating strategies of flow cytometric analyses of BMDCs.

DAPI^-^/CD11c^+^ population in BMDCs was gated to determine the expression levels of MHC class II and CD86.
